# Bewertung klinischer Arzneimittelprüfungen durch Ethikkommissionen: Änderungen durch die neue EU-Verordnung 536/2014 (CTR) und nötige Harmonisierungsmaßnahmen

**DOI:** 10.1007/s00103-022-03627-7

**Published:** 2022-12-06

**Authors:** Claudia Raffauf-Seufert, Guido Grass

**Affiliations:** 1 Ethikkommission der Friedrich-Alexander-Universität Erlangen-Nürnberg, Krankenhausstraße 12, 91054 Erlangen, Deutschland; 2 Ethikkommission der Medizinischen Fakultät der Universität zu Köln, Kerpener Str. 62, 50937 Köln, Deutschland

**Keywords:** Arzneimittelprüfung, Ethikkommission, Harmonisierung, Europäische Union, Clinical Trial Information System (CTIS), Clinical trial with medicinal products, Ethics committee, Harmonization, European Union, Clinical Trial Information System (CTIS)

## Abstract

Nach Wirksamwerden der Verordnung (EU) Nr. 536/2014 (Clinical Trials Regulation – CTR) des Europäischen Parlaments und des Rates am 31.01.2022 hat sich das Antragsverfahren für klinische Prüfungen grundlegend geändert. In diesem Beitrag werden die grundlegenden Verfahrensänderungen und die daraus für Deutschland und insbesondere für die Ethikkommissionen folgenden Veränderungen dargestellt. Auf die Harmonisierungsbestrebungen der Ethikkommissionen auf der EU-Ebene wird eingegangen.

Nach der neuen EU-Verordnung ist in Deutschland nur noch jeweils eine einzige Ethikkommission in die Genehmigung einer klinischen Arzneimittelprüfung eingebunden. Das bisherige Benehmensverfahren unter Einbeziehung mehrerer lokal zuständiger Ethikkommissionen wurde abgelöst. An dessen Stelle ist eine engere Zusammenarbeit der Ethikkommissionen mit den Bundesoberbehörden durch die Erstellung eines gemeinsamen Bewertungsberichts (Assessment-Report) sowie mit den anderen EU-Mitgliedsländern im Rahmen der Konsolidierung der jeweiligen länderspezifischen Anforderungen getreten. Der zuvor mit dem Benehmensverfahren einhergegangene regelmäßige gegenseitige Austausch der Ethikkommissionen hatte jedoch dazu beigetragen, Entscheidungskriterien, aber auch die Ermessensentscheidungen zu harmonisieren. Durch den Wegfall dieses Austauschs sind zum einen detailliertere Verfahrensempfehlungen erforderlich, aber auch regelmäßige andere Austauschmöglichkeiten, um die bereits bestehenden Harmonisierungen nicht nur beizubehalten, sondern auch weiter voranzutreiben. Der Arbeitskreis Medizinischer Ethik-Kommissionen in der Bundesrepublik Deutschland e. V. (AKEK) steht dazu als Vertretung der einzelnen Ethikkommissionen auch in intensivem Austausch mit Bundesoberbehörden, Antragstellern, anderen europäischen Ethikkommissionen und europäischen Institutionen.

## Abstract (en)

After Regulation (EU) No. 536/2014 (Clinical Trial Regulation, CTR) of the European Parliament and of the Council took effect on 31 January 2022, the application process for clinical trials fundamentally changed. This article describes the fundamental procedural changes and the resulting changes for Germany and, in particular, for the ethics committees. The harmonization efforts of the ethics committees at the EU level are discussed.

According to the new EU regulation, only one ethics committee is involved in the approval of a clinical trial of medicinal products in Germany. The previous consultation procedure involving several locally competent ethics committees has been replaced. Instead, there is now closer cooperation between the ethics committees and the federal authorities through the preparation of a joint assessment report as well as with the other EU member states as part of the consolidation of the respective country-specific requirements. The regular mutual exchange between the ethics committees that had previously accompanied the consultation procedure helped to harmonize the decision criteria but also the discretionary decisions. Due to the discontinuation of this exchange, more detailed procedural recommendations are required but also other regular exchange possibilities in order to not only maintain but also to advance harmonizations already in place. The Working Group of Medical Ethics Committees (AKEK), as the representative of the individual ethics committees, also maintains an intensive exchange with federal authorities, applicants, other European ethics committees and European institutions.

## Einleitung

Mit dem Wirksamwerden der Verordnung (EU) Nr. 536/2014 des Europäischen Parlaments und des Rates (Clinical Trials Regulation – CTR) am 31.01.2022 [1] hat sich das Antrags- und Bewertungsverfahren für klinische Arzneimittelprüfungen in Europa grundlegend geändert. Diese Änderungen und die Auswirkungen auf die Beteiligten sollen aus Sicht der Ethikkommissionen im Folgenden kurz dargelegt werden. Es soll aufgezeigt werden, bei welchen Änderungen ein Harmonisierungsbedarf besteht und wie dieser bereits angegangen wird.

Zunächst werden die grundlegenden Verfahrensänderungen durch die Einreichung des Antrags über das neue EU-Portal CTIS (Clinical Trials Information System) dargestellt. Anschließend wird auf die daraus für Deutschland resultierende Veränderung, insbesondere für die Ethikkommissionen eingegangen. Diese betreffen sowohl die Arbeitsweise der Ethikkommissionen untereinander als auch die nun engere Zusammenarbeit mit den zuständigen Bundesoberbehörden Bundesinstitut für Arzneimittel und Medizinprodukte (BfArM) und Paul-Ehrlich-Institut (PEI) sowie die neue gemeinsamen Bewertung der klinischen Prüfungen. Zum Schluss wird auf die Harmonisierungsbestrebungen der Ethikkommissionen auf EU-Ebene eingegangen, die sich jetzt neu durch die gemeinsame Bewertung der beteiligten EU-Länder ergeben und sich aktuell im Aufbau befinden.

## CTIS-Genehmigungsverfahren

**Figure 1:**
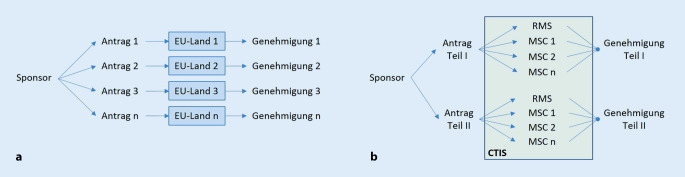

Durch die Einreichung der Antragsunterlagen über CTIS braucht ein Sponsor nur noch einen einzigen Antrag zu stellen und kann damit in einem Verfahren eine Genehmigung für eine klinische Prüfung mit der Zustimmung für alle geplanten Prüfzentren in der EU erhalten. Wie in Abb. 1a dargestellt, waren dazu bisher separate (zumeist noch papierbasierte) Anträge in allen an einer klinischen Prüfung beteiligten EU-Ländern notwendig, die jeweils in eigenen, unabhängigen Verfahren geprüft wurden. Dies führte nicht selten zu unterschiedlichen Nachfragen und Nachforderungen, was den Sponsor vor die Herausforderung stellte, die Studiendokumente entweder aufwendig zu harmonisieren oder länderspezifische Ergänzungen zu machen.

Das neue Antragsverfahren sieht nun im Rahmen der zentralen Einreichung zwei separate Teile vor (Abb. 1b). Teil I umfasst u. a. mit Prüfplan, Prüferinformationen und Nutzen-Risiko-Bewertung im weitesten Sinne das Studiendesign und die Prüfmedikation, so dass die Unterlagen hierzu nun EU-weit einheitlich sind. Teil II umfasst die landesspezifischen Unterlagen wie die Unterlagen für die Studienteilnehmer (in Landessprache) sowie die Unterlagen zu den Prüfzentren und Prüfern.

Insbesondere für den Antragsteller hat sich damit das Einholen einer Genehmigung für eine multinationale klinische Prüfung wesentlich vereinfacht. Das Verfahren sieht nun vor, dass die Nachfragen und Nachforderungen der jeweiligen EU-Länder vorab konsolidiert werden und diese auch gleichzeitig und gemeinsam vom Antragsteller zu beantworten sind. Für jedes Antragsverfahren wird nun ein berichterstattender Mitgliedstaat (Reporting Member State – RMS) bestimmt, dessen Aufgabe es ist, den Entwurf des Bewertungsberichtes (Draft Assessment Report) zu erstellen und die Anmerkungen aus den weiteren beteiligten Mitgliedstaaten (Member States Concerned – MSC) zusammenzufassen. Jeder Mitgliedsstaat kann daneben weitere Regelungen erlassen, wie das Bewertungsverfahren auch hinsichtlich der Einbindung der Ethikkommissionen auf Landesebene erfolgt.

## Genehmigungsverfahren in Deutschland

Mit Wirkung zum 26.01.2022 wurden die bisherigen Regelungen im deutschen Arzneimittelgesetz (AMG; [2]) und der GCP-Verordnung^1^ [3] aufgehoben. Bisher war ein separater Antrag auf Genehmigung bei der Bundesoberbehörde (BOB) und auf zustimmende Bewertung bei der zuständigen Ethikkommission notwendig. Beides waren 2 voneinander unabhängige Verfahren, die mit 2 separaten Bescheiden endeten. Auch hier konnte es durchaus vorkommen, dass unterschiedliche Nachforderungen durch die Bundesoberbehörde und die zuständige Ethikkommission gestellt wurden und es im Endergebnis zu unterschiedlichen Entscheidungen hinsichtlich Zustimmung und Versagung kam.

**Figure 2:**
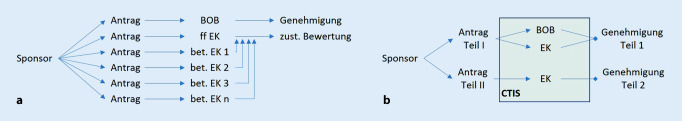

Im Falle von multizentrischen Studien musste der Antrag zudem auch bei allen für die einzelnen Prüfstellen zuständigen Ethikkommissionen eingereicht werden (Abb. 2a). Das sog. Benehmensverfahren, wie es detailliert in § 8 GCP‑V geregelt war, gibt es nun jedoch nicht mehr. Dieses sah bei multizentrischen Prüfungen vor, dass die Bewertung durch eine unabhängige interdisziplinär besetzte Ethikkommission erfolgte, die nach Landesrecht für den Leiter der klinischen Prüfung (LKP) zuständig war. Diese wurde als „federführende Ethikkommission“ bezeichnet (vgl. § 42 Abs. 1 AMG in der bis zum 26.01.2022 geltenden Fassung – AMGaf). Sie traf ihre Entscheidungen im Benehmen mit den „beteiligten Ethikkommissionen“. Diese prüften dabei insbesondere die Qualifikation der Prüfer und die Geeignetheit der Prüfstelle in ihrem Zuständigkeitsbereich (§ 8 Abs. 5 GCP-V).

Hinsichtlich der Einbindung der Ethikkommissionen im Rahmen der Bewertung nach CTR wurde in Deutschland festgelegt, dass die Ethikkommissionen für beide Teile des Antrags eine Bewertung abgeben (entsprechend § 40 Abs. 4 und 5 sowie § 41 Abs. 1 AMG). Die jeweils zuständige Bundesoberbehörde (BfArM oder PEI) beurteilt alle im Teil I zu bewertenden Aspekte und entscheidet über Genehmigung oder Versagung. Dabei muss die Stellungnahme der zuständigen Ethikkommission zu Teil I maßgeblich berücksichtigt werden. Für die Bewertung von Teil II ist die Bundesoberbehörde an die Stellungnahme der Ethikkommission gebunden (Abb. 2b).

Neu ist, dass zur Bewertung eines Antrags auf Genehmigung einer klinischen Prüfung nach CTR nur noch öffentlich-rechtliche Ethikkommissionen teilnehmen dürfen, die nach § 41a Abs. 2 bis 5 AMG registriert sind. Diese Registrierung haben jedoch derzeit nur 36 der 52 deutschen Ethikkommissionen beim BfArM beantragt [4]. Das hat zur Folge, dass bei angenommenen gleichbleibenden Antragszahlen sich die Arbeit auf weniger Ethikkommissionen verteilt. Gründe dafür, dass nicht alle Ethikkommissionen diesen Schritt mitgehen, sind vielfältig: Für die zum Teil nur knapp besetzten Geschäftsstellen bedeutet die Teilnahme am neuen Verfahren einen sehr viel höheren administrativen Aufwand als bisher, der u. a. durch die zum Teil sehr kurzen Fristen, das Arbeiten mit CTIS und den nun zu erstellenden Assessment-Report bedingt ist. Doch auch für die einzelnen ehrenamtlichen Mitglieder bedeutet dieser Konzentrationsprozess, dass mehr klinische Prüfungen von den einzelnen Mitgliedern begutachtet werden müssen als bisher, was zu einer zeitlichen Überlastung führen kann, gleichwohl kann die Reduzierung der Anzahl der im Prozess involvierten Ethikkommissionen möglicherweise zu einer Harmonisierung unter diesen beitragen.

Mit den Änderungen des AMG aufgrund des Inkrafttretens der CTR hat sich auch die Zuständigkeitsregelung der Ethikkommissionen geändert. Die Zuständigkeit richtet sich nun nicht mehr nach dem Leiter der klinischen Prüfung, sondern entsprechend § 41b AMG nach dem Geschäftsverteilungsplan der registrierten Ethikkommissionen.

Die Kriterien für die Erstellung des Geschäftsverteilungsplans sind in § 4 KBPV (Klinische Prüfung-Bewertungsverfahren-Verordnung; [5]) geregelt. Damit ist für alle Prüfzentren einer klinischen Prüfung in Deutschland nur noch eine der nach Landesrecht gebildeten Ethikkommissionen zuständig, unabhängig davon, ob eines der Prüfzentren in ihren lokalen Zuständigkeitsbereich fällt. Die Beteiligung lokaler Ethikkommissionen ist grundsätzlich nicht mehr vorgesehen.

Während der Übergangsfrist bis zum 31.01.2023 können beide Antragsverfahren (CTR bzw. AMGaf) alternativ verwendet werden. Bis Stand Ende August 2022 haben die Sponsoren nur verhalten von der Einreichung über CTIS Gebrauch gemacht und es wurden vorwiegend Anträge nach AMGaf eingereicht.

## Vor- und Nachteile des geänderten Antragsverfahrens für die Ethikkommissionen

In Bezug auf die Harmonisierung der Arbeit der Ethikkommission ist vor allem von Nachteil, dass durch den Wegfall des Benehmensverfahrens der unmittelbare Austausch zu einer konkreten klinischen Prüfung entfällt. Bislang liefen alle Stellungnahmen der beteiligten Ethikkommissionen bei der federführenden Ethikkommission zusammen und es oblag dieser, die Eingaben bei der Bewertung zu berücksichtigen. Die konsolidierten Nachforderungen und Bewertungen erhielten umgekehrt auch alle beteiligten Ethikkommissionen. Somit hat dieses Verfahren im Alltag zu einem gemeinsamen Lernprozess und der Bildung einheitlicher Bewertungsmaßstäbe beigetragen.

Dafür ist es ein großer Vorteil, dass nun die Bewertung der Ethikkommissionen gemeinsam mit den Bundesoberbehörden erfolgt. Dadurch, dass nach altem Recht 2 getrennte Anträge meist parallel liefen, gab es sowohl für die Bundesoberbehörden keinen Einblick in die Bewertung der Ethikkommissionen als auch umgekehrt. Auf die konkrete Zusammenarbeit wird im Folgenden noch ausführlicher eingegangen.

Weitere Vorteile des neuen Verfahrens sind, dass dieses hinsichtlich der gebotenen Schriftform über CTIS formaler und damit auch für Außenstehende transparenter ist. Ein direkter Kontakt mit dem Sponsor ist nicht mehr vorgesehen.

Nachteilig daran ist jedoch, dass nun kein anderer Weg zur Klärung von Details mehr zur Verfügung steht als über das CTIS und sogar kleine Defizite oder Unklarheiten zu einer Versagung führen könnten, sofern sie nicht bei der Beantwortung der Rückfragen (Requests for Information – RFI) ausgeräumt werden können. Die Klärung solcher Fragen war in der Vergangenheit einfacher möglich. Sie konnte durch die telefonische Rücksprache (seitens des Antragstellers oder der Ethikkommission) oder durch die Einladung eines Sponsorvertreters in die Ethikkommissionssitzung erfolgen. Von Vorteil war es auch, dass die lokal zuständigen Ethikkommissionen Kenntnisse über die lokalen Gegebenheiten der Prüfzentren und die dort ansässigen Prüfer besaßen.

Eine mögliche Einflussnahme des Sponsors durch die bewusste Auswahl der zuständigen Ethikkommission auf indirektem Weg durch die Wahl des Leiters der klinischen Prüfung ist nun aber auch nicht mehr möglich.

## Harmonisierung der Bewertung der Qualifikation von Prüfstellen und Prüfern

Unter dem Konzept der beteiligten Ethikkommissionen konnte es bei multizentrischen klinischen Prüfungen dazu kommen, dass je nach beteiligter Ethikkommission unterschiedliche Anforderungen an die Qualifikation der Prüfstellen und Prüfer innerhalb einer Studie gestellt wurden. Die Bestrebungen, die Bewertung der Qualifikation von Prüfer und Prüfzentren zu harmonisieren, müssen auch nach dem Wegfall der beteiligten Ethikkommissionen weitergeführt werden, damit auch studienübergreifend vergleichbare Anforderungen an die Qualifikation gestellt werden. Da den Ethikkommissionen nach dem neuen Verfahren die lokalen Gegebenheiten nicht mehr bekannt sind, ist eine Bewertung ausschließlich auf Basis der Antragsunterlagen möglich, was die Einreichung vollständiger und aussagekräftiger Unterlagen zwingend notwendig macht.

Zur einheitlichen Bewertung der regulatorischen Kenntnisse von Prüfer und Prüfstellen wurden schon vor vielen Jahren auch im Hinblick auf das Inkrafttreten der CTR in Zusammenarbeit der Bundesärztekammer (BÄK) und des Arbeitskreises der medizinischen Ethik-Kommission in der Bundesrepublik Deutschland e. V. (AKEK) die „Empfehlungen zur Bewertung der Qualifikation von Prüfern“ veröffentlicht, die regelmäßig aktualisiert werden. Die aktuelle Version vom 21.01.2022 geht bereits auf die Besonderheiten der CTR hinsichtlich der Prüfer und Mitglieder eines Prüfungsteams/einer Prüfergruppe ein [6]. Diese Empfehlungen beinhalten, welche regulatorischen Kenntnisse bei den Prüfern und ärztlichen Mitgliedern eines Prüfungsteams bzw. einer Prüfergruppe vorausgesetzt werden.

Die Anforderungen an die Inhalte der in den Empfehlungen genannten Kurse sind in separaten Veröffentlichungen – den sog. curricularen Fortbildungen – festgehalten. Diese sind der Grundlagenkurs für Prüfer und ärztliche Mitglieder eines Prüfungsteams [7], der Aufbaukurs für den Hauptprüfer, der eine Prüfergruppe bzw. ein Prüferteam/Prüfungsteam leitet [8], und der Auffrischungskurs [9], der alle 3 Kalenderjahre absolviert werden sollte und die aktuellen Rechtsänderungen berücksichtigt. Speziell für die Neuerungen der CTR gegenüber dem AMGaf wurde eine eigene curriculare Fortbildung entwickelt [10]. Ergänzend dazu wurden ebenfalls von der Bundesärztekammer zusammen mit dem AKEK FAQs zur curricularen Fortbildung für Prüfer und Mitglieder eines Prüfungsteams/einer Prüfergruppe veröffentlicht [11]. Diese dienen zur Erläuterung der curricularen Fortbildungen. Auf Basis dieser Empfehlungen fordern alle Ethikkommissionen dieselben Inhalte und Nachweise für die Bewertung der regulatorischen Kenntnisse der Prüfer.

Darüber hinaus haben die Ethikkommissionen gemeinsam mit der BÄK „Hinweise zur Bewertung der medizinischen Qualifikation der Prüfer und Mitglieder des Prüfungsteams“ erarbeitet und veröffentlicht [12]. Herausgearbeitet wurden 3 Hauptaspekte, nach denen ein Prüfer bewertet wird: die fachliche Qualifikation, die Einhaltung der regulatorischen Anforderungen und die Unparteilichkeit. Die Empfehlung befasst sich insbesondere mit der fachlichen Qualifikation, da die regulatorischen Anforderungen bereits in den Empfehlungen zur Bewertung der Qualifikation von Prüfern abgehandelt und „die Umstände, die die Unparteilichkeit beeinflussen könnten“, üblicherweise mit der Erklärung des (finanziellen) Interesses abgedeckt ist. Die Hinweise zu den fachlichen Anforderungen an die Qualifikation sind für alle Ethikkommissionen die Grundlage für die studienspezifische Bewertung der medizinischen Qualifikation der Prüfer und Mitglieder des Prüfungsteams.

Hinsichtlich der Bewertung der Eignung (Suitability) der Prüfstelle (Site) hat die EU Clinical Trials Expert Group das „Site Suitability Template“ entworfen [13], dessen Verwendung zur Prüfstellenbeschreibung im Rahmen des Antrags empfohlen wird. Dieses Template wurde vom AKEK durch Ausfüllhinweise und vorausgefüllte Textabschnitte ergänzt [14]. Die detaillierteren Erklärungen und der ergänzende Hilfetext sollen zum einen dem Sponsor helfen, die für die Ethikkommissionen zur Bewertung erforderlichen Angaben zu machen; diese dienen aber auch den Ethikkommissionen als Hilfestellung für eine einheitliche Bewertung. Die Template-Ergänzungen wurden auch in gemeinschaftlichem Übereinkommen mit den österreichischen Ethikkommissionen vorgenommen.

## Zusammenarbeit der Ethikkommission mit der Bundesoberbehörde bei der Bewertung von Teil I des Antrags

Die Bewertung von Teil I sieht nun einen gemeinsamen Assessment-Report der zuständigen Bundesoberbehörde und der zuständigen Ethikkommission vor. Der größte Vorteil für die inhaltliche Bewertung besteht darin, dass nun sowohl die Ethikkommissionen die zu beanstandenden Punkte der Bundesoberbehörden kennen als auch umgekehrt. Abweichende Meinungen sollten möglichst bereits während der Bewertungsphase geklärt werden. Somit erhält der Antragsteller mit der Nachforderung (RFI) bereits inhaltlich konsolidierte Nachfragen. In der Vergangenheit konnten unterschiedliche Änderungswünsche bei den 2 separaten Verfahren noch nachträgliche Änderungen erforderlich machen, um die Dokumente abzugleichen.

Vor dem Inkrafttreten der CTR wurde dazu ein „Gemeinsames Pilotprojekt von Bundesoberbehörden und Ethik-Kommissionen zur Bearbeitung von Anträgen klinischer Prüfungen mit Humanarzneimitteln entsprechend der EU‑V 536/2014 und unter gleichzeitiger Berücksichtigung der gesetzlichen Vorgaben von AMG und GCP-V“ ins Leben gerufen. Damit wurde ab 2015 sowohl den Sponsoren als auch der Bundesoberbehörde und den teilnehmenden Ethikkommissionen die Möglichkeit gegeben, erste Erfahrungen mit der Anwendung der CTR zu sammeln. Bis zum Auslaufen des Pilotverfahrens im September 2021 sind in etwa 350 Anträge bearbeitet worden.

Rückblickend hat das Pilotverfahren sicherlich maßgeblich zu einem guten gemeinsamen CTR-Start beigetragen. Ein großer Teil der Neuerungen – nämlich der gemeinsame Assessment-Report – war damit schon etabliert. Während der Zeit der Pilotverfahren wurden bereits Leitfäden für die gemeinsame Bearbeitung entwickelt, die lediglich an das tatsächliche Verfahren angepasst werden mussten. Beide Seiten hatten die Möglichkeit, die gegenseitige Arbeitsweise kennenzulernen. Und auch der sich während dieser Zeit etablierte regelmäßige offene Austausch hat dazu beigetragen, dass nach dem Start eine gute Basis für die Zusammenarbeit besteht.

Es hat sich gezeigt, dass die größte Hürde des gemeinsamen Verfahrens in den vielen formalen Feinheiten des CTIS liegen. Dazu wurde von der Arbeitsgruppe AG CTR/AMG des AKEK, die sich seit ihrer Gründung im November 2019 bereits mit Fragen und Handlungsempfehlungen zur Umsetzung der CTR beschäftigt, in Zusammenarbeit mit Vertretern der Bundesoberbehörden Leitfäden zur praktischen Anwendung von CTIS im Rahmen eines Bewertungsverfahrens für die Ethikkommissionen entwickelt. Der weiterhin regelmäßig stattfindende Austausch der Ethikkommissionen mit den Bundesoberbehörden und der Ethikkommissionen untereinander trägt wesentlich zur Harmonisierung der Arbeit der Ethikkommissionen bei, da konkrete Abläufe besprochen und Best-Practice-Erfahrungen geteilt werden.

## Harmonisierungsbestrebungen der europäischen Ethikkommissionen

Eine neue Dimension der Harmonisierung ergibt sich aus der durch die CTR geforderten gemeinsamen Bewertung von Anträgen durch die beteiligten EU-Mitgliedsstaaten. Das bisher gelebte föderale Prinzip wurde aus Sicht der Ethikkommission von der Bundesebene auf die europäische Ebene gehoben. So erfahren die Ethikkommissionen nun einen bisher nie dagewesenen Austausch mit den europäischen Nachbarn, sei es als RMS oder MSC. Es gilt nun, neue Sichtweisen in die eigene Arbeit zu integrieren. Bereits jetzt ist es spürbar, wie europaweit das Interesse von Ethikkommissionen zunimmt, sich über die jeweiligen Sichtweisen einerseits auszutauschen und andererseits Wege der Harmonisierung zu suchen. Immer mehr Mitgliedsländer beteiligen sich an der „Ethics Committee Advisory Group“ der Expert Group on Clinical Trials (CTEG). Diese berät die CTEG insbesondere zu Verfahrensfragen, die Teil II des Antrags betreffen sowie jene Aspekte von Teil I, die von den Ethikkommissionen zu bewerten sind. Gleichwohl befindet sich die Institutionalisierung der Beteiligung von Ethikkommission an Entscheidungsprozessen in europäischen Gremien gerade erst im Entstehen.

Der Assessor’s Round Table wurde von der Europäischen Kommission als informelles Forum des Austausches der Personen eingerichtet, die an der Bewertung klinischer Prüfungen durch die Mitgliedsländer beteiligt sind. Die Ethikkommission betreffende Themen finden dort nun ebenso einen Raum wie behördlicherseits zu bewertende Aspekte; aus mehr Mitgliedsstaaten als in anderen Gremien nehmen dort Vertreter der Ethikkommission die Gelegenheit zur Diskussion war.

Nicht in allen Mitgliedsländern gibt es ähnlich gefestigte Organisationsstrukturen des Austausches von Ethikkommissionen wie dies in Deutschland mit dem AKEK der Fall ist, was sich in der heterogenen Zusammensetzung des European Network of Research Ethics Committees (EUREC) niederschlägt [15]. Das Netzwerk EUREC möchte nationale Verbände von Forschungsethikkommissionen auf europäischer Ebene zusammenführen und die nationalen Ethikkommissionen bei der Zusammenarbeit mit anderen europäischen Gremien unterstützen. Jedoch sind die Aufgaben und Organisationsformen der Ethikkommissionen in Europa sehr verschieden. Während beispielsweise in den Niederlanden die Aufgaben der zuständigen Behörde von der Ethikkommission wahrgenommen werden oder in Italien die zuständige Ethikkommission am Ministerium für Gesundheit angesiedelt ist und diesem untersteht [16], erfolgt die Bewertung in anderen Ländern, wie beispielsweise Belgien oder Deutschland, von unabhängigen Ethikkommissionen. Auch durch diese strukturelle Heterogenität bedingt kommt es teilweise zu unterschiedlichen Erfahrungen und Sichtweisen, deren Harmonisierung wohl noch einige Zeit in Anspruch nehmen wird.

Zugleich kann jedoch nicht unerwähnt bleiben, dass die engere Verzahnung mit behördlichen Tätigkeiten auch eine Gefahr für die Unabhängigkeit der Ethikkommissionen darstellen kann. Mit Sorge mussten hier tatsächliche und versuchte Einflussnahmen teils auch auf inhaltliche Aspekte seitens der Europäischen Kommission gesehen werden.

## Fazit

Die Änderung des Antragsverfahrens für klinische Prüfungen ist für alle Beteiligten Neuland. Aufgrund der bisher von den Sponsoren verhaltenen Nutzung der Antragseinreichung über CTIS blieb den Beteiligten Zeit, sich mit dem Verfahren und der Verwendung des komplexen Antragsportals CTIS, das von einer fehlerfreien und komfortablen Anwendung noch entfernt ist, weiter vertraut zu machen.

Durch Wegfall des Benehmensverfahrens fehlt ein wichtiges Instrument der Kommunikation zwischen den Ethikkommissionen in Deutschland, das bisher zur Harmonisierung beigetragen hat. Durch den Zusammenschluss der Ethikkommissionen im AKEK wird es aber weiterhin den Dialog der deutschen Ethikkommissionen untereinander geben. Gerade auf formaler Ebene hat dieser bereits, wie bei der Bewertung der Qualifikation der Prüfstellen und Prüfer, zu einer Harmonisierung geführt. Mit regelmäßigen Tagungen und Online-Meetings wird weiterhin aktiv der Austausch gefördert, um inhaltliche Fragen zu diskutieren und nach Möglichkeit einheitliche Bewertungskriterien zu definieren.

Auch die gemeinsame Bewertung mit den Bundesoberbehörden wird zur Harmonisierung der inhaltlichen Bewertung beitragen, wobei der Input aus der aktuellen Wissenschaft und der klinischen Praxis durch die multidisziplinäre Expertise der Ethikkommissionen erhalten bleibt.

Die Zusammenarbeit auf europäischer Ebene stellt eine besondere Chance dar, auch im Bereich der ethischen Bewertung in Europa einheitlichere Standards zu entwickeln. Die Mitgestaltung dieser Harmonisierungsprozesse bedeutet für die Ethikkommission eine neue Herausforderung, aber auch große Kraftanstrengung. Die Mitwirkung in entsprechenden Gremien und das dort erwartete Engagement sind mit der Tätigkeit als Ehrenamt oder im Nebenamt teils schwer zu realisieren.

Nicht zuletzt wird es auch Aufgabe der Ethikkommissionen sein, dafür Sorge zu tragen, dass bei der europäischen Harmonisierung die vordringliche Aufgabe des Schutzes der betroffenen Person und zukünftiger Patientinnen und Patienten nicht wirtschaftlichen Interessen nachgeordnet wird.

Der größte Vorteil des harmonisierten Antragsverfahrens ist gewiss, dass in definierter Zeit eine Genehmigung zur Durchführung einer multizentrischen klinischen Prüfung in der EU erhalten werden kann, was den Forschungsstandort EU sicherlich stärkt.

## Footnotes

1 GCP-V: Verordnung über die Anwendung der Guten Klinischen Praxis bei der Durchführung von klinischen Prüfungen mit Arzneimitteln zur Anwendung am Menschen.
